# A rare form of discoid lupus erythematosus as a rosacea and angiofibroma: A case report

**DOI:** 10.1002/ccr3.2611

**Published:** 2019-12-19

**Authors:** Fatemeh Mokhtari, Zakiye Ganjei

**Affiliations:** ^1^ Department of Dermatology Skin diseases and leishmaniasis research center Isfahan University of medical science Isfahan Iran; ^2^ Resident of Dermatology Department of Dermatology Skin diseases and leishmaniasis research center Isfahan University of medical science Isfahan Iran

**Keywords:** angiofibroma, discoid lupus erythematosus, rosacea

## Abstract

The rare presentation of DLE can be rosacea‐/angiofibroma‐like lesions which should be considered in patients with red‐pink to skin‐color papules with flushing and photosensitivity.

## Graphical abstract

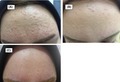

## 1 INTRODUCTION

The present study reports a 26‐year‐old female with pink‐to‐red and skin‐colored papules on the face with photosensitivity and easy flushing that clinically mimicked rosacea and angiofibroma without response to routine treatments while skin biopsies confirmed cutaneous discoid lupus erythematosus. The rare presentation of DLE can be rosacea‐/angiofibroma‐like lesions.

Lupus erythematosus is a disease with specific clinical and histological characteristics with cutaneous presentations. Cutaneous features of lupus may be presented concurrently with or without its systemic manifestations.^1^

According to the Düsseldorf classification, cutaneous lupus erythematosus (CLE) is classified into four subgroups including acute cutaneous lupus erythematosus (ACLE), subclinical erythematosus (SCLE), chronic erythematosus (CCLE), and intermittent erythematosus (ICLE).^2^

Discoid lupus erythematosus (DLE), hyperkeratotic discoid lupus erythematosus syndrome, lupus erythematosus and lichen planus overlap syndrome, mucosal lupus erythematosus, chilblain lupus erythematosus, and lupus erythematosus panniculitis/profundus are presentations classified as chronic types of cutaneous lupus erythematosus.^2^, ^3^

Discoid lupus erythematosus is the most common form of CCLE,^3^, ^4^, ^5^ often associated with facial involvement in the form of red indurated plaques with distinct margins and crust causing pigmentation change, scar formation, and eventually atrophy.^3^, ^5^

Rosacea is a chronic recurrent skin disease with various forms of presentations including papules, pustules, erythema, and telangiectasia that affect central areas of the face (cheeks, chin, nose, and middle frontal area) and sometimes ocular involvement.^6^

Dome‐shaped papules on the skin from forehead to the bridge of the nose and cheeks are a usual manifestation of angiofibroma that is mostly found in tuberous sclerosis.^7^

Dessinioti et al presented that a red face is not always equal to rosacea.^6^

In confirmation of their study, we presented a patient with cutaneous presentations mimicking rosacea and angiofibroma due to the lesions limited to the center of the face and eventual diagnosis of DLE.

## 2 CASE PRESENTATION

A 26‐year‐old female referred to the dermatology clinic with a chief complaint of pink‐reddish lesions on the face.

The patient mentioned photosensitivity since 5 years ago. She experienced redness changes on sunlight‐exposed parts of the body (eg, face and hands) and then healed following some days of being unexposed.

Furthermore, by progression of the disease, she explained that pink‐to‐red lesions appeared on the central parts of the face and forehead following sun exposure. However, these lesions spontaneously improved following a period of sun avoidance.

Since 2 years ago, she was encountered with more intensified lesions as they developed to most of the facial areas including the nose, cheeks, periorbital area, forehead, and chin, which did not disappear spontaneously. Moreover, she complained of numerous repeated daily flushes as the new presentation of her disease.

Her previous medical history showed evidence of no disease except a history of mild acne during puberty and adolescence. There was no previous history of drug use or a similar presentation in her family.

**Figure 1:**
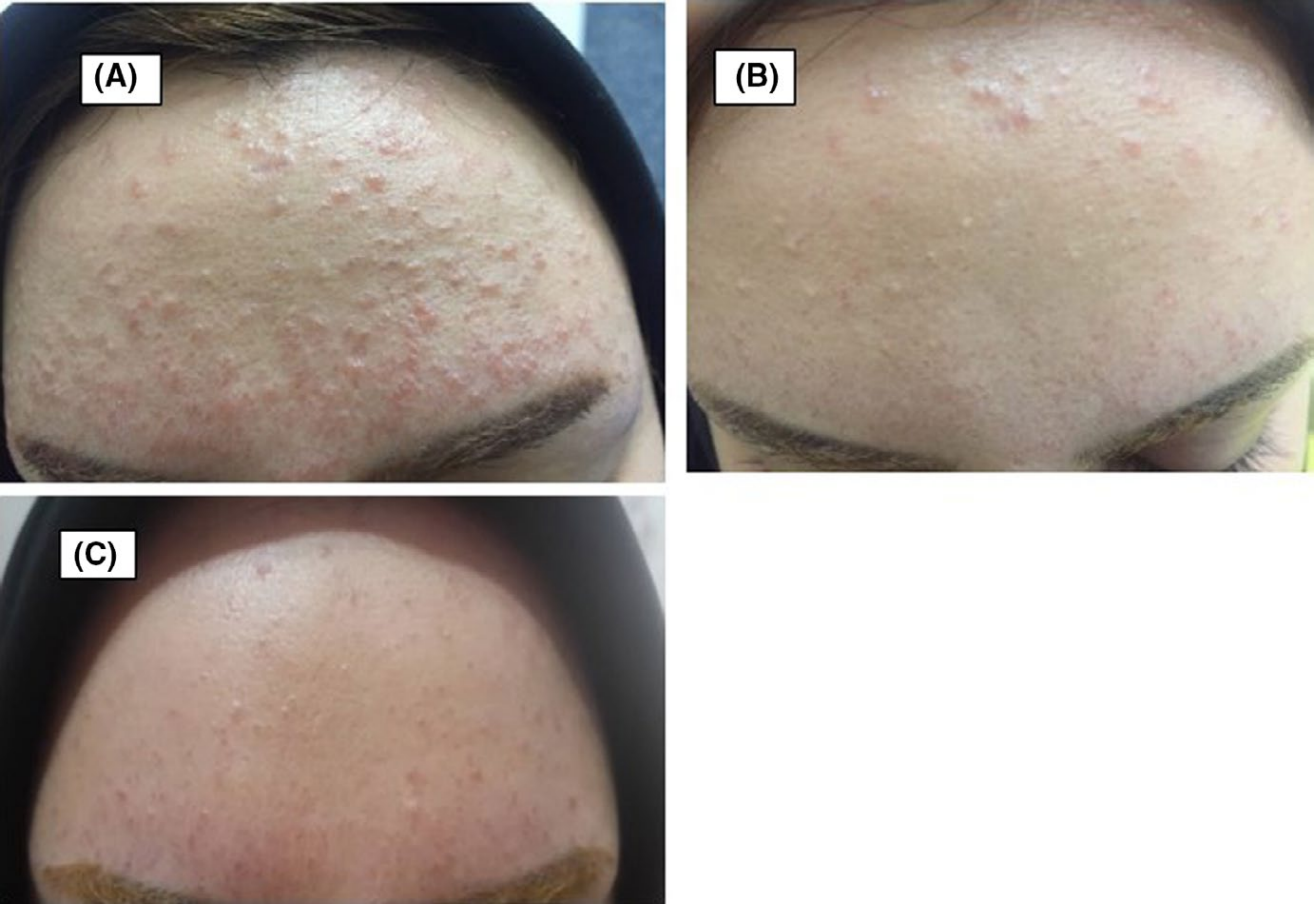
Discoid lupus erythematosus. A, red‐pink to the skin‐colored papules on the forehead. B, Remission of lesions following a month of treatment. C, Remission of lesions following 6 mo of treatment

In the patients' facial examinations, red‐pink to skin‐color papules were visible, occasionally on the red background, eyelids excluded (Figure 1A). In addition, scars due to previous acne were detected. No comedone, pustule, telangiectasia, atrophy, or edema was found on her face.

Further physical examination revealed no other skin lesions or systemic dysfunctions.

In addition, she presented an initial diagnosis of rosacea in other outpatient clinics treated by systemic antibiotics and topical metronidazole gel. She was also advised to protect her face using continuous sunscreen. In spite of a year of treatment, even with systemic isotretinoin, no recovery or even improvement was found by the patient.

Then, a presumptive diagnosis of angiofibroma was made and laser treatment was performed for some of the lesions that were all irresponsive to laser therapy.

Due to treatment response failure, she lost her confidence and failed in performing daily chores.

Thereafter, she referred to outpatient dermatology clinic of AL‐Zahra Hospital, affiliated at Isfahan University of Medical Sciences. We performed biopsies from lesions with differential diagnoses of rosacea, angiofibroma, Demodex folliculorum, rosacea granulomatous, and acne miliaris.

**Figure 2:**
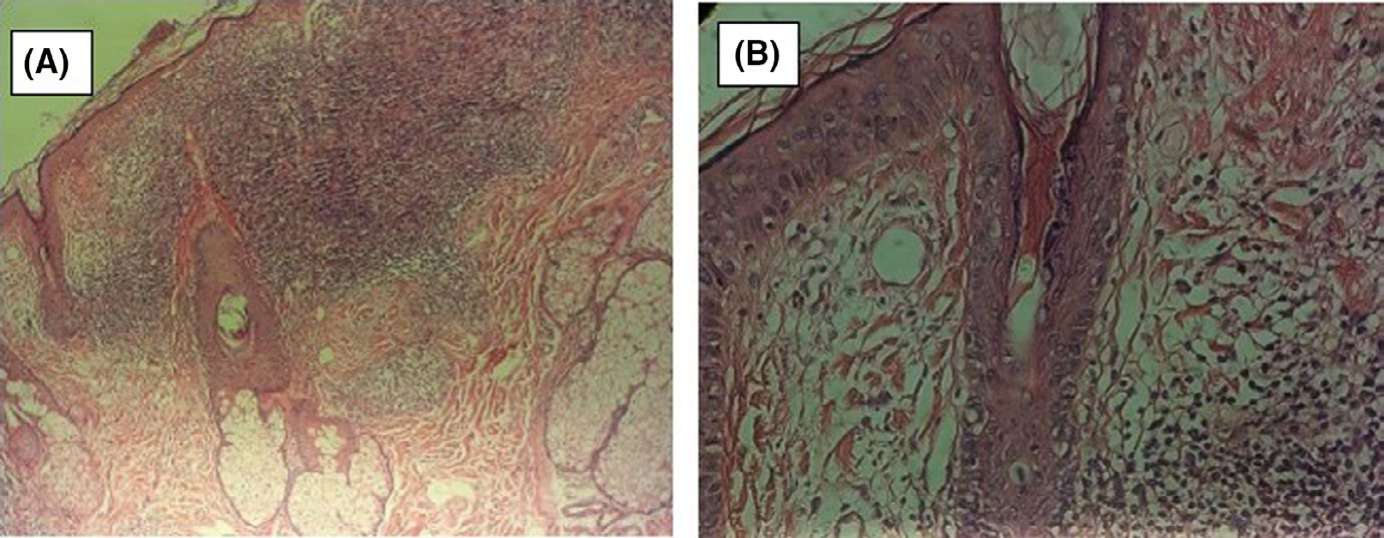
Basketweave orthokeratosis with epidermal atrophy and focal degeneration of the basal layer and severe infiltration in the upper and middle dermis, interstitial tissue around the vessels and the hair follicles along with small epithelioid aggregation; it was seen as an ill‐defined granuloma without caseous necrosis. (A, H&E staining with 10 times magnification, B, H&E staining with 40 times magnification)

In the histopathology of facial lesions biopsies, we found basket weave orthokeratosis, epidermal atrophy, focal degeneration of basal layer, severe infiltration in the upper and middle dermis, interstitial tissue around the arteries, and follicles of hair with small epithelioid cell aggregation as ill‐defined granuloma (Figure 2). According to mentioned histopathological reports of biopsies, the diagnosis of DLE was made.

Therefore, systemic blood tests such as complete blood count (CBC/diff), erythrocytic sedimentation rate (ESR), anti‐nuclear antibody (ANA), anti‐double‐stranded DNA (anti‐ds DNA), blood urea nitrogen (BUN), creatinine (Cr), and urine analysis (U/A) were requested for her, which all revealed normality.

Eventually, the patient was diagnosed as DLE and treated with daily mometasone cream and pimecrolimus 1% cream. She was also advised to avoid sunlight and to routinely use sunscreen.

Within a month, noticeable recovery of the lesions was detected (Figure 1B), remission of lesions was obvious following 6 months of treatment, and she could retrieve her lost confidence and returned to normal life.

## 3 DISCUSSION

Erythematous lesions spread following sun light exposure is a characteristic feature of lupus erythematosus,^8^ and unusual lesions such as red‐pink to skin‐colored papules without crust are not considered as usual features of lupus erythematosus lesions.

Lupus lesions are usually manifested as erythematous‐purple plaques with shells, or butterfly rashes (malar rush) and scarring may occur in discoid plaque‐type lesions of DLE.^1^ However, our patient was primarily diagnosed with rosacea and angiofibroma due to her facial lesions. Features including long duration of the disease, absence of scar, and hypopigmentation/depigmentation distracted physicians from the diagnosis of DLE.

On the other hand, red‐pink popular lesions with a slight background of erythema and flushing are consistent with the classic diagnosis of acne rosacea, while the absence of pustule, telangiectasia and ocular complication and failure to respond to classic rosacea treatment in special questioned this diagnosis.^6^, ^8^

As a manifestation of tuberous sclerosis, angiofibroma was the other differential diagnosis made for the patient.^9^ Although the patient's lesions were somewhat similar to angiofibroma lesions, further physical examinations showed no signs of tuberous sclerosis.

Since multiple pseudo‐angiofibroma may be detected in patients with no symptom of tuberous sclerosis, the patient should be screened for multiple endocrine neoplasia type 1 (MEN1). Thus, biopsies should be obtained from the lesions, and in case of angiofibroma, tuberous sclerosis, and diagnosis confirmation, the patient should be screened for MEN1.^10^

Such a rare manifestation of discoid lupus erythematosus has not been mentioned except in the Rook's Textbook of Dermatology stating that 7.5% of patients with DLE may present lesions similar to rosacea. These patients have nodular red lesions on the nose, cheeks, forehead, and sometimes chin with diffuse erythema. Flushing is a usual presentation of these patients.^11^ We should emphasize that the patient in this study presented reddish‐pink skin papules, not nodular red lesions. Furthermore, she did not present facial erythema but had scattered erythema in some part of her face with slight redness. In fact, it can be concluded that DLE diagnosis through obtaining biopsies prior to empirical therapy initiation should be considered for presentation of rosacea‐ or angiofibroma‐like lesions in addition to photosensitivity and flushing.

We were unable to perform direct immunofluorescence but it can provide additional diagnostic elements.^12^

In case of eventual DLE diagnosis, treatment with topical agents can successfully manage the lesions without antimalarial agent requirement.

## 4 CONCLUSION

One of the rare patterns of DLE can be rosacea‐angiofibroma which should be considered in patients with red‐pink to skin‐color papules with flushing and photosensitivity.

## CONFLICT OF INTEREST

None declared.

## AUTHOR CONTRIBUTIONS

FM: visited the patient, interpreted and reviewed the patient data, revised the manuscript and performed patient follow‐up. ZG: was responsible for visiting the patient and medical care, performed biopsy, drafted the initial manuscript, and provided photographs.
